# Detection of Inter-Turn Short-Circuit Faults for Inverter-Fed Induction Motors Based on Negative-Sequence Current Analysis

**DOI:** 10.3390/s25154844

**Published:** 2025-08-06

**Authors:** Sarvarbek Ruzimov, Jianzhong Zhang, Xu Huang, Muhammad Shahzad Aziz

**Affiliations:** School of Electrical Engineering, Southeast University, Nanjing 210096, China; s.k.ruzimov@seu.edu.cn (S.R.); huangxuee@seu.edu.cn (X.H.); 233209910@seu.edu.cn (M.S.A.)

**Keywords:** fault diagnosis, induction machine, inverter, negative-sequence current analysis, short-circuit fault

## Abstract

Inter-turn short-circuit faults in induction motors might lead to overheating, torque imbalances, and eventual motor failure. This paper presents a fault detection framework for accurately identifying ITSC faults under various operating conditions. The proposed method integrates negative-sequence current analysis utilizing wavelet-based filtering and symmetrical component decomposition. A fault detection index to effectively monitor motor health and detect faults is presented. Moreover, the fault location is determined by phase angles of fundamental components of negative-sequence currents. Experimental validations were carried out for an inverter-fed induction motor under variable speed and load cases. These showed that the proposed approach has high sensitivity to early-stage inter-turn short circuits. This makes the framework highly suitable for real-time condition monitoring and predictive maintenance in inverter-fed motor systems, thereby improving system reliability and minimizing unplanned downtime.

## 1. Introduction

Induction motors (IMs) are widely employed in industrial systems due to the advantages of high durability, low cost, and simple structure [1]. Despite their mechanical robustness, IMs remain vulnerable to electrical faults, particularly inter-turn short-circuit (ITSC) faults in the stator windings. This type of fault, if left undetected, can lead to localized heating, magnetic flux imbalance, reduced efficiency, and ultimately motor failure [2,3].

ITSC faults in IMs are predominantly caused by insulation failure between nearby stator turns resulting from a confluence of thermal, electrical, mechanical, and environmental stressors that accumulate over time [4]. Extended temperature exposure, especially in areas without sufficient cooling, causes the degradation of insulation via microcracking and embrittlement, with the lifespan of the insulation generally reducing by half for every 10 °C increase beyond its designated class rating [5]. The electrical stress from contemporary inverters intensifies insulation deterioration due to large dV/dt switching transients, which can surpass 1000 V/μs and provoke partial discharges within the turn insulation, particularly near the motor terminals, where voltage reflections are significant [6]. Mechanical variables, including rotor imbalance, bearing degradation, and resonance-induced vibrations, result in slot wedge loosening and conductor displacement, leading to abrasion and subsequent insulation failure [7]. Moreover, manufacturing defects—such as inconsistent coil tensioning, inadequate vacuum pressure impregnation (VPI), and insufficient resin curing—can create localized weak points susceptible to premature failure under operational stress [7]. Environmental pollutants, such as moisture, dust, and corrosive gases, markedly diminish surface insulation resistance, resulting in surface tracking and dielectric breakdown, especially in humid or chemically hostile environments [8].

Various methods have been established in recent decades for the detection of ITSC faults in IMs. Early methodologies concentrated on model-based observers [9], thermal analysis [10], and vibration- or speed-based techniques [11,12]. Model-based schemes yield fault-sensitive residuals; however, they necessitate precise motor parameters and are frequently susceptible to model uncertainty [9]. Thermal monitoring is effective for tracking long-term degradation; however, it is hindered by slow response and issues with spatial resolution, particularly under varying load conditions [10]. Analysis of vibration and rotor speed can reveal mechanical imbalances resulting from stator asymmetries; however, these methods typically necessitate supplementary sensors and are affected by external mechanical disturbances [11,12]. Motor current signature analysis (MCSA) has emerged as a preferred diagnostic strategy to address these limitations, owing to its non-intrusive characteristics and appropriateness for real-time implementation. In MCSA, the analysis of negative-sequence current (NSC) has proven effective in identifying asymmetries caused by ITSC faults [13]. Several methods based on NSC have been developed. The authors of [14] introduced a phasor-based compensation method that addresses the fault-induced NSC component by correcting voltage imbalances, sensor inaccuracies, and machine asymmetries. This approach, although highly accurate, requires precise modeling and calibration, which restricts its adaptability in dynamically changing environments. In [15], researchers enhanced this approach by experimentally isolating NSC disturbances from fault components in low-power motors; however, the method is still susceptible to unmodeled noise sources. A semiempirical compensation model [16] was proposed to enhance robustness in non-stationary operating conditions, addressing motor nonlinearities, load variations, and supply imbalances via a power decomposition technique. Nonetheless, its accuracy is significantly influenced by the reliability of empirical models, which may deteriorate in transient situations. The authors of [17] introduced a PC-based NSC monitoring system featuring rapid-response and compensation mechanisms; however, its evaluation was restricted to small-scale motors in laboratory environments, thereby constraining its industrial applicability. The authors of [18,19] examined fault evolution modeling using sequence impedance and transformation-based models, achieving effective fault localization, but with increased computational complexity. In [20], an NSC method without sensors utilizing off-diagonal impedance matrix elements was introduced, which demonstrated significant robustness without the need for additional hardware; however, it necessitates stable operating conditions to prevent false positives. Despite advancements, practical NSC-based diagnosis encounters challenges in inverter-fed environments, where switching harmonics and spectral noise frequently obscure the fundamental NSC signal [21]. Conventional signal extraction techniques, including fast Fourier transform (FFT) and Park’s vector analysis (PVA), demonstrate limited robustness when applied to variable-frequency drives (VFDs) and transient load shifts [22,23].

Furthermore, neural networks have been utilized in the diagnosis and prognosis of electric motors. In [24], multilayer feedforward neural networks (FNNs) were utilized to identify short circuits between coils in direct-on-line permanent magnet synchronous motors (PMSMs). This study contrasted statistical metrics derived from steady-state currents with frequency-domain analysis. A comparable study is presented in [25], where the discrete wavelet transform (DWT) was utilized on a motor’s line currents to derive descriptive characteristics based on the energy of the resultant components, and a comparison was made between the performance of multilayer perceptron and recurrent neural networks. In [26], support vector machines (SVMs) and convolutional neural networks (CNNs) were employed, utilizing voltage and quadrature current data to train the algorithms. In all instances, precision levels surpassed 99%, although it was observed that CNNs necessitate a greater volume of data than support vector machines. In [27,28], the authors offered extensive assessments on the advancements in fault detection and diagnosis in electrical machines utilizing artificial intelligence. These compilations emphasized critical components, including the application of indicators derived from frequency-domain analysis using FFT or wavelet transform to identify diverse faults in electrical machinery.

On the other hand, recent studies have addressed these limitations by introducing advanced signal processing methods, including empirical mode decomposition (EMD) [29], Hilbert–Huang transform (HHT) [30], and variational mode decomposition (VMD) [31], which have shown enhanced effectiveness in isolating fault-related features. These approaches frequently involve significant computational costs, mode-mixing challenges, or sensitivity to parameter adjustments, which limits their use in real-time industrial settings. Wavelet-based signal processing has demonstrated significant potential for non-stationary signal analysis, attributed to its multi-resolution and time-frequency localization capabilities [32,33]. Integrating symmetrical component decomposition with wavelet filtering provides an effective method for selectively extracting fault-relevant negative-sequence components, even under significant inverter-induced disturbances.

In addition to detection of faults, an ability to identify faulty phases in an IM is crucial for accurate diagnostics and focused maintenance. A possible method for faulty phase localization is analyzing the phase angle of the fundamental component of the NSC. Although the method based on the phase angle of the NSC has been used for fault detection [14,15], the method has not been studied for localization of faulty phases. Paper [14] emphasizes the efficacy of NSC phasor analysis in identifying shorted turn faults in IMs. However, it specifically omits any discussion on utilizing the phase angle of the NSC to identify the fault stator phase, which is a critical aspect of comprehensive motor diagnostics. In [15], the authors researched NSC phasor analysis, which was utilized exclusively to extract a fault-induced NSC for detection rather than identifying the specific faulty stator phase. The phase angle of the NSC was neither employed nor addressed for fault-phase localization, signifying that the method focuses on detection rather than comprehensive diagnostic categorization.

Many inverter topologies have been invented to fulfill the performance, efficiency, and reliability requirements of electrical machine drives [34]. Conventional two-level inverters have become common due to their simplicity. Nonetheless, they experience significant harmonic distortion and switching losses at elevated voltage levels. To address these constraints, multilayer inverter (MLI) configurations such as neutral point clamped (NPC), flying capacitor (FC), and cascaded H-bridge (CHB) have been developed. The NPC inverter is widely utilized in medium-voltage applications owing to its enhanced voltage waveform quality and reduced dv/dt stress. Nonetheless, it necessitates several clamping diodes, hence augmenting conduction losses and complexity. In paper [35], the authors introduced an innovative multilevel neutral point (MNP) inverter design that eliminates all clamping diodes through the use of both mono- and bidirectional switches. This method not only streamlines the hardware architecture but also improves fault tolerance, as the inverter can function in a reduced mode in the event of a switch failure. Moreover, the architecture facilitates a low-switching-frequency control method, rendering it appropriate for high-reliability and low-loss applications in power electronics.

This paper introduces an innovative method for ITSC fault detection and locating faulty phases in inverter-fed IMs, integrating wavelet-based filtering with NSC analysis to tackle the issues identified above. A fault detection index (FDI) to measure fault severity and enable the prompt identification of ITSC defects is proposed. The phase angle of the NSC is a key metric in locating faulty phases. The suggested method was experimentally validated under diverse load and speed situations, exhibiting enhanced sensitivity and robustness relative to current NSC-based diagnostic techniques.

Our key research objectives were:

1. Development of a diagnostic framework that combines DWT with NSC analysis, specifically tailored for inverter-fed motor systems.
2. Formulation of an FDI that quantitatively assesses ITSC severity across different operational conditions.
3. Localization of the faulty phase utilizing the phase angle of the fundamental component of the NSC.
4. Thorough experimental validation in authentic industrial settings, emphasizing the method’s excellence in early fault identification and real-time applicability.

This paper is organized as follows. Section 2 details the modeling of ITSC faults and the proposed detection methodology, including signal processing techniques and the framework. Section 3 presents the experimental setup and discusses the findings. Section 4 discusses key indicators of the fault detection framework. Section 5 concludes the paper.

## 2. Methodology

### 2.1. Analytical Model of Stator ITSC Fault

**Figure 1:**
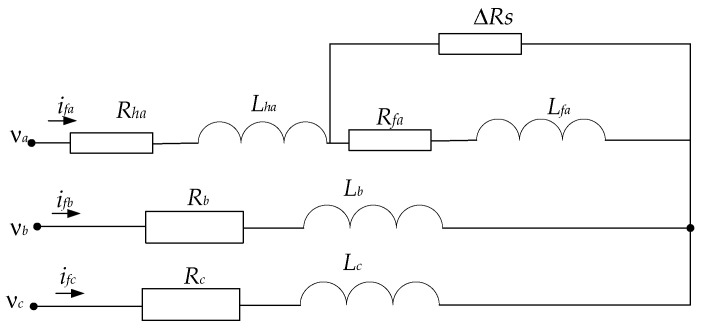
Representation of stator winding with ITSC fault in phase A.

An ITSC fault in an IM affects the electrical parameters, flux linkages, and symmetries and results in vibrations, harmonics, and NSCs. Supposing a fault occurs in a fraction *μ* of stator turns, additional resistive and inductive imbalances will happen, as shown in Figure 1.

Figure 1 depicts an analogous circuit model of a three-phase IM experiencing an ITSC fault in phase A. The upper branch denotes the fault phase, whereby the winding is partitioned into a functional segment characterized by resistance *R_ha_* and inductance *L_ha_*, and the defective portion is indicated by *R_fa_* and *L_fa_*. An extra resistor D*R_s_* is connected in parallel to the shorted turns to replicate the fault pathway. The latter two phases (B and C) are presumed to be healthy and are represented using lumped resistance *R_b_*_,*c*_ and inductance *L_b,c_*. The input voltages and line currents are associated with each phase. This form is frequently employed to simulate fault processes, assess fault severity, and validate detection algorithms in electrical machine diagnostics.

For a healthy IM, the voltage equations on the *dq*-axis are as follows:

(1) $$\left\{\begin{array}{c} v_{ds}=R_{s}i_{ds}+\frac{d\lambda_{ds}}{dt}-\omega_{e}\lambda_{qs}\\ v_{qs}=R_{s}i_{qs}+\frac{d\lambda_{qs}}{dt}+\omega_{e}\lambda_{ds} \end{array}\right.$$

where *i_ds_* and *i_qs_* are the stator currents in the *dq*-frame, *R_s_* is the stator resistance, *ω_e_* is the electrical angular frequency. *λ_ds_* and *λ_qs_* are stator flux linkages in the *dq*-frame, represented thus:

(2) $$\left\{\begin{array}{c} \lambda_{ds}=L_{s}i_{ds}+L_{m}i_{dr}\\ \lambda_{qs}=L_{s}i_{qs}+L_{m}i_{qr} \end{array}\right.$$

where *L_S_* is the stator inductance, *L_m_* is the mutual inductance, and *i_dr_*, *i_qr_* are the rotor currents.

For an IM with an ITSC fault in a certain phase, the voltage equations are modified as follows:

(3) $$\left\{\begin{array}{c} v_{fds}=(R_{s}+\Delta R_{s})i_{fds}+\frac{d}{dt}(L_{fs}i_{fds}+L_{fm}i_{fdr})-\omega_{e}(L_{fs}i_{fqs}+L_{fm}i_{fqr})\\ v_{fqs}=(R_{s}+\Delta R_{s})i_{fqs}+\frac{d}{dt}(L_{fs}i_{fqs}+L_{fm}i_{fqr})+\omega_{e}(L_{fs}i_{ds}+L_{fm}i_{fdr}) \end{array}\right.$$

where Δ*R_s_* is the additional resistance due to the ITSC fault and *L_fs_* and *L_fm_* are the inductance and mutual inductance, respectively. The inductance of the faulty IM is:

(4) $$L_{fs}=L_{s}-\Delta L_{s}$$

where Δ*L_s_* is the deviation due to the short-circuit fault:

(5) $$\Delta L_{s}=\mu^{2}L_{s}(1+\beta_{f}e^{-\gamma_{f}t})$$

where *μ* is the shorted turn fraction, where 0 < *μ* < 1. *β_f_* and *γ_f_* are empirical constants that quantify degradation due to heating, magnetic distortion, and nonlinear progression of the fault. Combining Equations (4) and (5), *L_fs_* is modified as:

(6) $$L_{fs}=L_{s}(1-\mu^{2}-\mu^{2}\beta_{f}e^{-\gamma_{f}t})$$

Mutual inductance *L_fm_* of the faulty IM can be expressed as:

(7) $$L_{fm}=L_{m}(1-\mu^{2})$$

The resistance modifications due to fault evolution are described as:

(8) $$\Delta R_{s}=\mu R_{s}\frac{1+\alpha_{f}}{1-\mu }$$

where *α_f_* is the empirical constant for degradation.

### 2.2. Negative-Sequence Current Analysis Under Inverter Harmonics

The negative-sequence current is extracted using symmetrical component transformation:

(9) $$\left[\begin{array}{c} I_{0}\\ I_{1}\\ I_{2} \end{array}\right]=\frac{1}{3}\left[\begin{array}{ccc} 1 & 1 & 1\\ 1 & a & a^{2}\\ 1 & a^{2} & a \end{array}\right]\times \left[\begin{array}{c} I_{a}\\ I_{b}\\ I_{c} \end{array}\right]$$

where $a=e^{j\frac{2\pi }{3}}$, and *I_a_*, *I_b_*, and *I_c_* are the three-phase currents.

When the IM is fed by a PWM inverter, the inverter will introduce harmonics to the machine that can obscure the ITSC fault signatures. In order to explicitly account for these inverter-induced distortions, a sophisticated mathematical framework of combining wavelet transform and symmetrical component analysis is needed to adequately separate fault-induced negative-sequence currents from harmonic distortions to increase the accuracy and reliability of ITSC fault detection. This integration technique guarantees that the NSC is free of harmonic distortions to increase the reliability and accuracy of fault detection in the presence of complex non-ideal conditions.

#### 2.2.1. Discrete Wavelet Transform Decomposition

To isolate the harmonic distortions, a discrete wavelet transform (DWT) is applied. We apply a level-*J* orthogonal DWT to each phase current of the IM [36]:

(10) $$\left\{A_{J}[n],D_{J}[n],D_{J-1}[n],\ldots ,D_{1}[n]\}=DWT(I_{abc}[n];\sigma ,\rho \right)$$

where *σ* is the mother wavelet, *ρ* is a scaling function, *A_J_* is the level-*J* approximation (low-pass) coefficients, *D_J_* is the detail (high-pass) coefficient at scale *J* of the wavelet coefficients, *n* is sample index. The selection of *J* is:

(11) $$J=[\log_{2}(\frac{f_{s}}{f_{H\max}})]$$

where *f_s_* is the sampling frequency and *f_Hmax_* the highest switching harmonics.

This ensures that harmonics appear in detail bands *D*_1_ to *D_J_*.

The Daubechies 8 (db8) wavelet was chosen as the mother wavelet for the DWT because of its equilibrium between temporal and spectral localization, as well as its efficacy in detecting the transient current spikes characteristic of ITSC defects. Daubechies wavelets are orthogonal and compactly supported, rendering them especially appropriate for the decomposition of non-stationary signals. The symmetric padding extension technique was employed to mitigate border artefacts. The decomposition level *J* was determined utilizing the sample frequency and the highest-observed switching harmonic, so ensuring that all pertinent harmonic components were encompassed within the detail bands. A comparison with alternative wavelet basis functions, such as Haar, db4, and db20, showed that db8 provides enhanced harmonic identification and smoother reconstruction while minimizing spectral leakage. Lower-order wavelets, such as Haar, exhibited inadequate frequency resolution, and higher-order wavelets, like db20, increased processing demands without markedly enhancing fault isolation [37]. The symmetric boundary extension method was employed to prevent artificial discontinuities at the signal edges, hence ensuring the robustness of the decomposition. Consequently, the chosen design (db8, optimal *J*, symmetric padding) offers an advantageous balance between accuracy and efficiency.

A soft-threshold *λ_J_* is estimated by Stein’s unbiased risk estimate (SURE) for each detail band *D_J_*:

(12) $$\lambda_{J}=\arg\min SURE(D_{J},\lambda )$$

(13) $${\tilde{D}}_{J}[n]=\left\{\begin{array}{c} \mathrm{sgn}(D_{J}[n])(|D_{J}[n]|-\lambda_{J}),|D_{J}[n]|>\lambda_{J},\\ 0,otherwise. \end{array}\right\}$$

The soft threshold preserves transient fault spikes while suppressing steady harmonics. The harmonic components are then reconstructed using the inverse DWT based on the thresholded detail bands:

(14) $$I_{H}[n]=IDWT(0,{\tilde{D}}_{J}[n],\ldots ,{\tilde{D}}_{1}[n])$$

Subtracting the harmonic components from the total current yields a cleaner signal:

(15) $$I_{c}[n]=I_{abc}[n]-I_{H}[n]$$

#### 2.2.2. Fault Detection Index

A robust and reliable fault indicator based on the detection and quantification of ITSC faults in IMs that remains sensitive to fault-induced asymmetries while being immune to power inverter harmonics is required. In order to accomplish this, the FDI is proposed to improve accuracy and robustness in locating an ITSC fault.

(16) $$FDI=k_{1}\frac{\left|I_{2}(t)\right|}{I_{2H}}$$

where *k*_1_ represents weighting factors obtained by experimental optimization and *I*_2*H*_ is the NSC of a healthy motor.

*k*_1_ serves to increase or decrease the normalized NSC magnitude, ensuring reliable fault indication performance under diverse dynamic situations. The selected value is an appropriate balance between sensitivity (true-positive rate) and specificity (false-alarm resistance), as demonstrated in experimental validation to assure generalizability. The optimization of *k*_1_ was conducted by an offline grid search method with a dataset comprising diverse operational conditions. In this work, *k*_1_ = 0.27 is tuned experimentally based on the dataset captured.

The term $k_{1}\left|I_{2}(t)\right|/I_{2H}$ is a normalized magnitude of the NSC, which is a direct indicator of electrical asymmetry in the stator windings. This is important because ITSC faults result in imbalances in the stator circuit with the result of increasing negative-sequence components.

The FDI is a reliable fault detection parameter that distinguishes genuine fault-induced NSC deviations from non-fault disturbances. This approach significantly improves the real-time fault detection accuracy of IMs, especially in a distorted inverter environment. In this work, this metric is validated through experimental testing under controlled and industrial operating conditions to assess its capability of detecting ITSC faults of different severity.

#### 2.2.3. Interpolation-Based FDI Threshold Setting

Although the resultant FDI values differ under different scenarios, a singular universal threshold is necessary to facilitate real-time fault identification without condition-specific adjustments. We employ an interpolation-based method to establish a universal threshold, utilizing the linear trend identified in FDI growth as fault severity increases. Linear interpolation is employed to fit the FDI values at specified fault severity levels for each speed–load pair.

(17) $$FDI(\mu )=FDI(\mu_{i})+\frac{\mu -\mu_{i}}{\mu_{i+1}-\mu_{i}}\times [FDI(\mu_{i+1})-FDI(\mu_{i})],\mu \in [\mu_{i},\mu_{i+1}]$$

Equation (17) allows for the estimation of interpolated FDI values at detailed severity levels across various speed and load conditions. We define the universal FDI threshold to ensure comprehensive fault coverage as follows:

(18) $$FDI_{TH}=\underset{\mu >\mu_{\min}}{\min}[FDI(\mu )]$$

This threshold ensures complete separation and reduces the probability of false alarms. This single threshold, once implemented, facilitates robust fault detection across all motor speeds, loads, and fault severities, eliminating the necessity for case-by-case calibration.

#### 2.2.4. Identification of Fault Location

Alongside identifying ITSC faults in IMs, it is imperative to precisely isolate faulty phases to provide data for repair decisions and minimize system downtime. An excellent method for accomplishing this entails examining the phase angle of the fundamental component of the NSC, obtained from the symmetrical component decomposition of the stator line currents. In a balanced and healthy IM functioning under nominal conditions, the NSC component is ideally insignificant, and its phasor angle holds no diagnostic relevance. When an ITSC fault occurs in a stator winding, the resultant electrical asymmetry generates a specific negative-sequence component, with its phasor angle systematically varying based on the affected phase. This phenomenon occurs due to the directional imbalance in current flow resulting from uneven impedance among the three phases. Utilizing FFT to isolate the fundamental-frequency NSC component from real-time current data enables precise computation of angular displacement, hence facilitating accurate localization of the faulty phase.

To obtain the magnitude and phase of an NSC, the NSC is extracted at a fundamental frequency using FFT. The equation is:

(19) $$I_{2}(f_{fund})=\left|I_{2}\right|e^{j\theta_{I2}}$$

*θ_I_*_2_ is simply the angle of this complex number:

(20) $$\theta_{I2}=\arg(I_{2}(f_{fund}))$$

In an ideally balanced three-phase system, the stator windings are electrically separated by 120°, as are the supply voltages and currents. An ITSC fault in one phase disrupts symmetry, causing the resultant asymmetry to manifest as NSC. The negative-sequence system is characterized by a collection of phasors that possess identical magnitude and frequency to the original system, but rotate in the opposite direction (clockwise for a negative system). Therefore: (a) in the event of a fault in phase A, the resultant NSC phasor will have a particular orientation in relation to the system reference; (b) if the fault occurs in phase B, the resultant NSC phasor will shift approximately 120°; (c) the fault in phase C results in an additional 120° displacement approximately.

### 2.3. Fault Detection Framework

**Figure 2:**
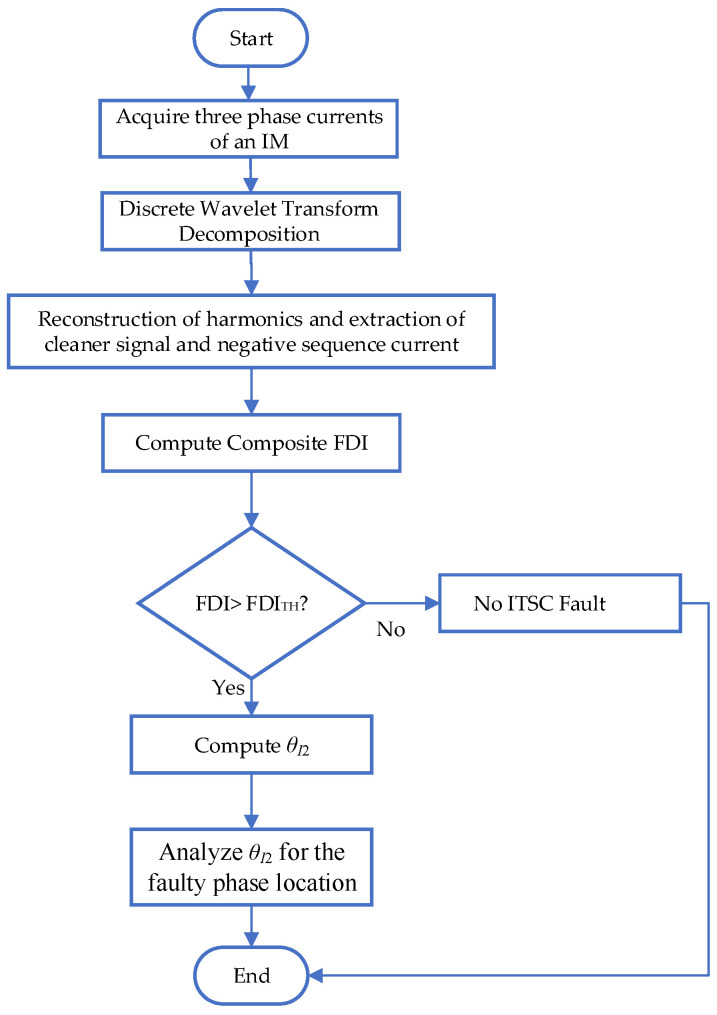
Flowchart of proposed fault detection framework.

The flowchart of the method is shown in Figure 2. The proposed framework introduces a groundbreaking analytical foundation for ITSC fault modeling by integrating multiple layers of realism and precision. Central to this innovation is a parameterized mathematical representation of the ITSC fault that not only localizes the fault region but also quantifies the severity of winding degradation through scalable variables. Unlike traditional models that treat faults as binary events, this approach dynamically maps fault evolution and its impact on the system’s electromagnetic profile. Additionally, the model explicitly incorporates the effects of PWM inverter-induced harmonic distortions, allowing for accurate simulation of real-world industrial motor conditions, where such harmonics often mask early fault indicators. The framework also presents a novel decoupling methodology that isolates the fault-induced NSC components from those generated by harmonic artifacts, thereby improving diagnostic clarity. Through a combination of state-space equations and parametric tables, the model links fault progression to measurable current asymmetries, enabling precise identification of fault onset and severity. Moreover, it introduces the FDI, which synergizes time-domain waveform distortions and frequency-domain spectral shifts, overcoming the diagnostic limitations inherent in single-feature detection systems. This comprehensive and adaptive structure forms the foundation for a more robust, interpretable, and scalable ITSC fault detection system.

## 3. Experimental Setup and Validations

**Figure 3:**
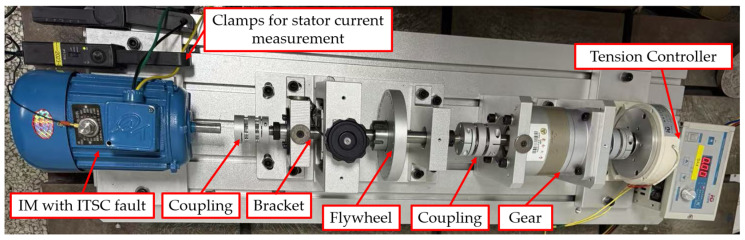
Complete laboratory-based experimental setup.

The experimental validation of ITSC fault detection was carried out using a fully instrumented motor test bench, shown in Figure 3, designed for monitoring current waveforms, collecting real-time performance data, and inducing controlled faults under various load and speed conditions. The setup integrates a three-phase squirrel cage IM coupled to a magnetic powder brake, a digital tension controller, an oscilloscope, an industrial inverter drive, and a monitoring PC running dedicated motor control software (Figure 3).

In the energy conversion chain, an inverter produced by Chinese company Inovance Technology (model MD600S, Shenzhen, China) was used to power the IM. The inverter functions at a switching frequency of 5 kHz and utilizes insulated-gate bipolar transistors (IGBTs) as the switching components, selected for their rapid operation and thermal resilience under varying load circumstances [34]. Motor speed regulation is accomplished using a scalar voltage–frequency (V/f) control methodology, which sustains a consistent volts-per-hertz ratio to maintain magnetic flux and guarantee stable torque production throughout the speed spectrum. The inverter output is directly linked to the motor terminals without any filtering stages. This direct connection enables the incorporation of high-frequency components, including current harmonics and asymmetries, in the measured signals. The negative-sequence components are particularly significant, as they function as the principal indicators for identifying ITSC faults under the proposed diagnostic framework.

The magnetic powder brake, linked via a precision shaft coupling, allowed for adjustable loading on the motor shaft. Its torque was regulated using the HD800 tension controller, Shenzhen Hpmont Technology Co., Ltd., Shenzhen, China This controller provided accurate braking force by adjusting the magnetic field strength applied to the brake, enabling the study of ITSC behavior under different loading conditions.

For signal acquisition and fault signal monitoring, a Yokogawa DLM2024, Shanghai, China, mixed-signal oscilloscope was used. Connected via differential probes, it captured stator current and voltage waveforms with high temporal resolution. This was essential for analyzing negative-sequence current patterns and identifying harmonic components during different fault scenarios. In addition, the experimental setup involved current measuring with Hall-effect clamps, commonly employed in inverter-fed motor diagnostics. Hall-effect clamps can measure both AC and DC components, rendering them appropriate for detecting low-frequency fault signals and steady-state imbalances. Their extensive bandwidth and electrical isolation render them optimal for precisely monitoring current aberrations caused by inverter switching. Conversely, Rogowski coil clamps—due to their non-intrusive nature and exceptional flexibility—demonstrate superior efficacy in detecting high-frequency transient components, including harmonics and switching noise. When utilized alongside an oscilloscope, these clamps facilitate thorough current monitoring, allowing for accurate extraction of negative-sequence components and fault-related signs.

The dataset captured a wide range of operating scenarios by varying three primary parameters: frequency and load conditions. Three supply frequencies—10 Hz, 30 Hz, and 50 Hz—were selected to reflect practical motor supply variations. Fault levels were 1% (0.01), 7% (0.07), and 15% (0.15), simulating different severities of turn-to-turn shorting within the stator windings. For the test bench, the IM with an ITSC fault was designed as: 1% of fault severity in phase A, 7% of fault severity in phase C, and 15% of fault severity in phase B, and fault severity was controlled with three switches, one for each. Load conditions were simulated at three levels, 0%, 50%, and 100%, to evaluate how mechanical stresses influence fault signatures under real-world operations. Each experiment was saved in.xlsx format, containing high-resolution time-series waveforms of three-phase stator currents. In total, the dataset comprises approximately 150 files, making it a robust and diverse benchmark for developing and validating ITSC fault detection models.

It is crucial to note that this study did not use any simulation models. Using the test bench shown in Figure 3, the diagnostic technique was put into practice and verified directly through hardware-based experimentation. The experimental test bench depicted in Figure 3 was built for this study, and the parameters of the main equipment are shown in Table 1.

**Figure 4:**
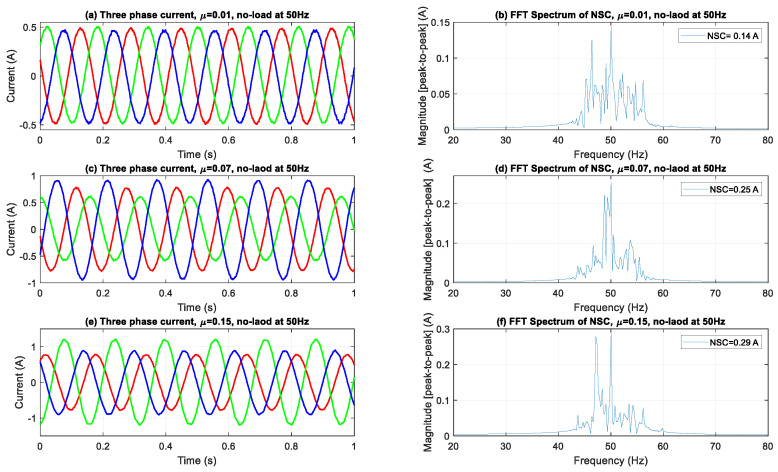
Three-phase current waveforms and FFT spectra of NSC under no load.

**Figure 5:**
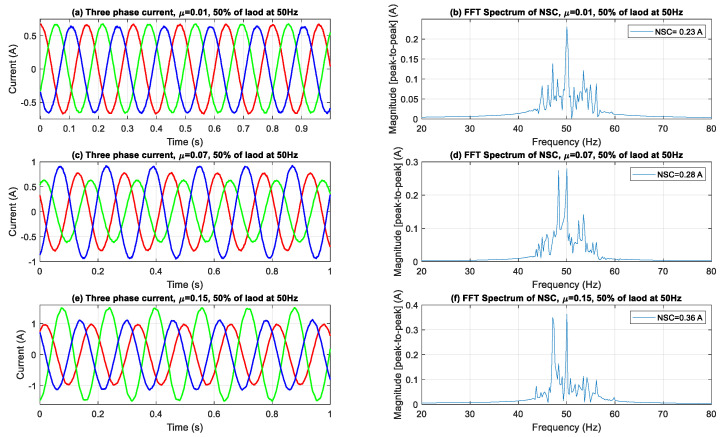
Three-phase current waveforms and FFT spectra of NSC under 50% load.

**Figure 6:**
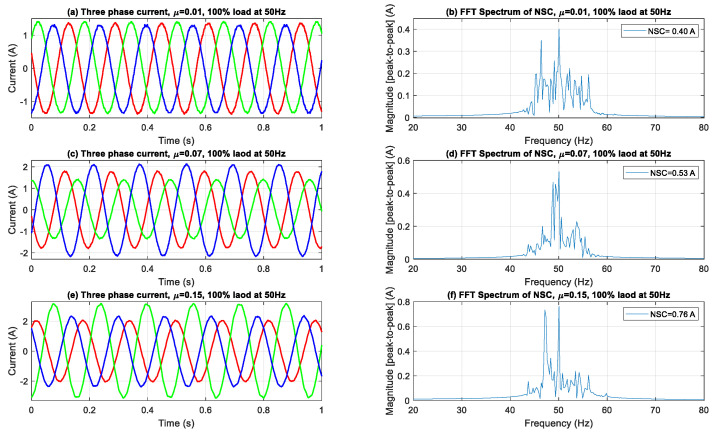
Three-phase current waveforms and FFT spectra of NSC under 100% load.

Figure 4, Figure 5 and Figure 6 illustrate the evolution of three-phase current waveforms and their associated NSC spectra under different ITSC fault severities and load situations. During the no-load operation depicted in Figure 4, the currents initially display excellent sinusoidal characteristics with negligible asymmetry. As fault severity escalates, amplitude imbalances and phase mismatches progressively manifest, signifying the onset of stator winding problems. Despite the lack of mechanical strain, frequency-domain analysis indicates substantial spectrum alterations, characterized by a marked increase in the NSC fundamental component (50 Hz) and the emergence of sideband harmonics. This signifies that NSC analysis is exceptionally responsive to early-stage defects, able to identify tiny asymmetries that could go unnoticed in conventional current monitoring methods.

When the motor experiences a 50% load, as depicted in Figure 5, the current amplitudes rise, exacerbating the occurrence of phase imbalances in the time domain. The NSC spectra exhibit a more significant rise in both the fundamental and sideband components, indicating enhanced fault-induced asymmetries under partial loading. The emergence of sidebands, mainly due to rotor slip modulation interacting with the supply frequency, becomes more pronounced, suggesting that loading conditions can amplify the visibility of fault signs in the spectral domain.

Figure 6 illustrates that during full-load operation, the effects of the ITSC faults are significantly amplified. Significant amplitude imbalances and waveform distortions are observed as fault severity escalates. The NSC spectra reveal a marked increase in the fundamental component, with the severe fault scenario (NSC ≈ 0.76 A) displaying predominant spectral peaks and greatly amplified sideband components. The proliferation of these harmonics highlights the escalating interplay between stator asymmetry and rotor dynamics under elevated torque requirements, thus enhancing the spectrum data accessible for diagnostic analysis.

In Figure 4, Figure 5 and Figure 6, the sideband components detected at frequencies ±2*sf* from the fundamental frequency indicate modulation effects resulting from ITSC asymmetry in loaded conditions. The sidebands observed are indicative of rotor-slot harmonics interacting with stator field distortions due to fault-induced imbalance, leading to periodic torque pulsations and current modulations. The frequency offset of 2*sf*, which is twice the slip frequency, corresponds with established theoretical models regarding slot harmonics influenced by stator asymmetry in squirrel-cage IMs. The proposed method employs DWT filtering to specifically attenuate the high-frequency switching components inherent in inverter-fed systems. The impact of residual inverter harmonics near ±2*sf* is negligible, allowing for the conclusion that the observed sidebands are primarily due to fault-related electromagnetic interactions rather than artifacts from switching.

The aggregated outcomes at all load levels validate the reliability and sensitivity of NSC-based spectral analysis in identifying and measuring ITSC failures. The continuous increase in both the primary NSC component and its related side-bands with heightened fault severity and load levels underscores the efficacy of this method for early detection, severity assessment, and real-time condition monitoring of IMs across diverse operating conditions.

### 3.1. Fault Detection Index and Threshold Setting

This subsection discusses the FDI and the establishment of thresholds based on the interpolation method.

**Figure 7:**
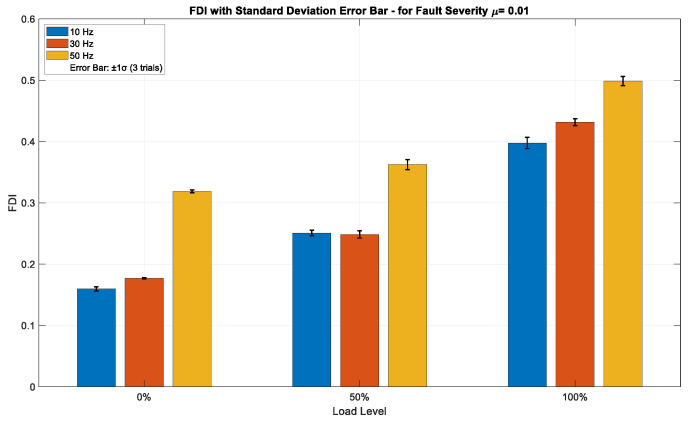
FDI values for fault severity *μ* = 0.01 across varying load and frequency conditions, with ±1σ error bars from three independent trials.

**Figure 8:**
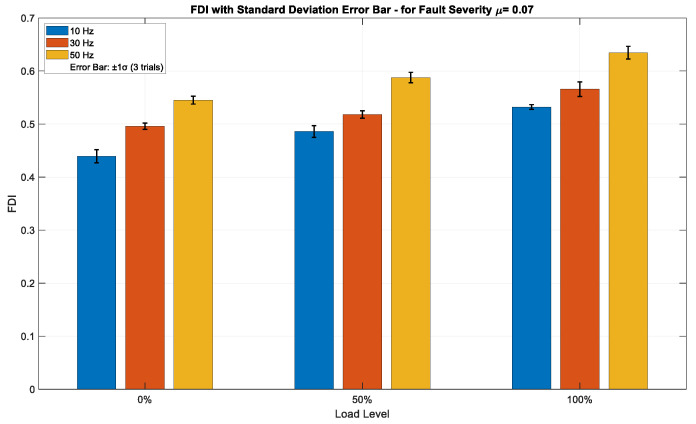
FDI values for fault severity *μ* = 0.07 across varying load and frequency conditions, with ±1σ error bars from three independent trials.

**Figure 9:**
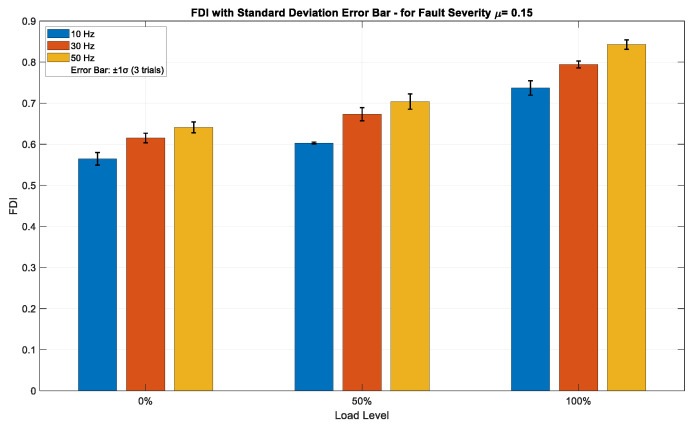
FDI values for fault severity *μ* = 0.15 across varying load and frequency conditions, with ±1σ error bars from three independent trials.

Figure 7, Figure 8 and Figure 9 illustrate the FDI values for fault severities of *μ* = 0.01, 0.07, and 0.15, respectively, across different load conditions and excitation frequencies, with standard deviation (±1σ) error bars obtained from three independent trials per data point. The FDI exhibits a distinct monotonic increase across all three severity levels in relation to both load and frequency, underscoring the index’s sensitivity to operational stress. At the minimal fault severity level (*μ* = 0.01), Figure 7 depicts a visible, but moderate increase in FDI values corresponding to both load and frequency. The FDI escalates from approximately 0.16 at 10 Hz and 0% load to about 0.50 at 50 Hz and 100% load, indicating that the method can effectively identify ITSC faults even at low severity. Error bars representing ±1σ, derived from three independent acquisitions, remain narrow across all operational points—typically within ±0.01–0.015—signifying high repeatability and minimal measurement noise. This implies that the FDI is stable and statistically dependable even in the initial stages of fault progression. The insignificant magnitude of FDI at this level further confirms its sensitivity threshold without having false alarms. As fault severity rises to *μ* = 0.07, shown in Figure 8, the FDI demonstrates a higher gradient over both load and frequency. Values fluctuate from roughly 0.45 under low-stress settings to nearly 0.63 under high-stress operation (50 Hz, 100% load). This middle fault level exhibits an enhanced, but coherent reaction, sustaining a continuous trend of FDI growth amid operational stress. Error bars are minimal (±0.01–0.02), exhibiting a tiny rise in deviation at elevated load–frequency levels, as anticipated due to more dynamic and nonlinear motor behavior. The consistency seen in the trials verifies that the FDI is both responsive to fault propagation and statistically resilient to transient variations in the data collection method. At the highest severity level (*μ* = 0.15), Figure 9 illustrates the most significant escalation in FDI, with values fluctuating between about 0.57 and 0.84 across the analyzed conditions. The trend is consistently monotonic and strongly associated with rising load and frequency, confirming the method’s ability to detect severe fault signs. The difference in amplitude among frequencies is particularly noticeable under full load, with 50 Hz regularly producing the highest FDI measurements. Despite the marginal expansion of the standard deviation bars (up to ±0.025), the intra-condition variability stays comfortably within the anticipated ±1σ range, hence maintaining interpretability. The findings indicate that the approach preserves accuracy and dependability even in severe fault situations, rendering it appropriate for real-time monitoring in industrial settings.

Table 2 presents detailed values of FDI for various fault severities under different operational conditions. Increased frequency and load result in higher fault severity, which corresponds to elevated FDI, as demonstrated in Table 2. Table 3 summarizes the FDI range after the threshold is set for different fault severities.

The proposed composite FDI and threshold-setting method successfully detected even very low fault severity under all load and frequency combinations. Fault detection became stronger and more stable as fault severity increased. This makes the detection system reliable for real-time fault monitoring under varying industrial conditions.

### 3.2. Localization of the Faulty Phase

**Figure 10:**
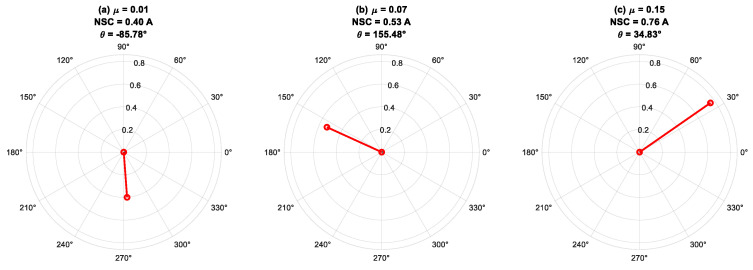
Phase angles of negative-sequence current for different fault severity at *f* = 50 Hz under 100% load.

**Figure 11:**
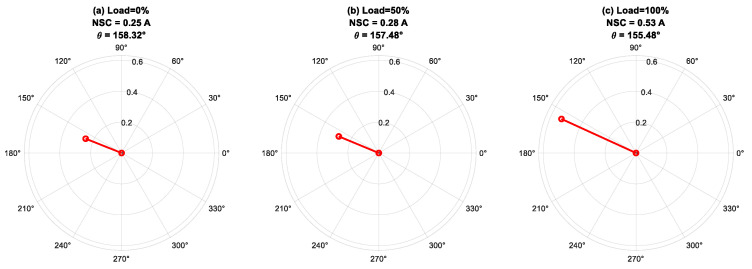
Phase angle of negative-sequence current for fault severity *μ* = 0.07, *f* = 50 Hz under varying load conditions: 0%, 50%, and 100%.

**Figure 12:**
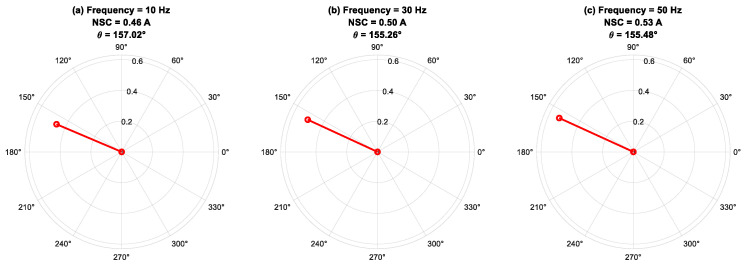
Phase angle of negative-sequence current for fault severity *μ* = 0.07, 100% of load under different frequencies.

In this subsection, the phase angle *(θ*_I2_*)* of the fundamental component of an NSC is analyzed using FFT for different fault severities under the varying load and frequency combinations shown in Figure 10, Figure 11 and Figure 12.

Figure 10 illustrates the phase-angle characteristics of the fundamental component of the NSC at 50 Hz under full-load conditions, with differing fault severities applied across various stator phases of the IM. The findings indicate that the NSC phase angle (*θ*_I2_) is predominantly influenced by the fault’s phase position rather than its intensity. Faults in phases A, B, and C generate phase angles approximately centered at 0°, 120°, and −120°, respectively, aligning with theoretical predictions. The observed fluctuations in NSC magnitude indicate alterations in fault severity, but minor discrepancies in phase angle are ascribed to intrinsic machine asymmetries and measurement noise. This investigation underscores the efficacy of NSC phase-angle monitoring as a diagnostic instrument for precise fault-phase identification, with NSC magnitude offering additional insights into fault propagation and severity.

Figure 11 displays the phase-angle response of the fundamental component of the NSC at 50 Hz, assessed under a constant fault severity of *μ* = 0.07 throughout load levels of 0%, 50%, and 100%. The analysis indicates that the phase angle of the NSC is predominantly unaffected by load fluctuations, highlighting its significant reliance on the location of the faulty phase rather than the loading conditions. The consistent stability of the phase angle under different mechanical loads demonstrates the reliability of *θ*_I2_ as an effective signal for fault-phase localization. Minor angular fluctuations observed at various load levels are ascribed to non-ideal factors, including magnetic saturation, and intrinsic asymmetries in the stator winding and supply system. The NSC magnitude’s sensitivity to load variations, along with the phase angle’s relative insensitivity, highlights the complementary functions of both parameters in delivering thorough diagnostic insights for early defect detection and severity evaluation in an IM.

Figure 12 presents the phase-angle behavior of the fundamental component of the NSC at 100% load, with a constant fault severity of *μ* = 0.07, across different supply frequencies. The findings indicate that the NSC phase angle exhibits stability throughout the examined frequency range, highlighting the primary impact of the fault-phase position on *θ*_I2_, as opposed to fluctuations in supply frequency. Minor variations in phase angle observed with frequency changes are ascribed to frequency-dependent phenomena, including magnetic saturation, skin effect, and core losses, which result in slight asymmetries in the electromagnetic response of the machine. The robustness of the NSC phase angle in relation to frequency variations supports its use as a dependable fault-phase indicator across various operating conditions. Concurrently, the variations in NSC magnitude reflect the combined influences of fault severity and frequency-dependent machine behavior. This analysis demonstrates the effectiveness of NSC-based phase-angle monitoring for reliable, frequency-insensitive fault localization within condition monitoring systems.

Based on analysis of *θ*_I2_, the following criteria are set to identify fault location:

(21) $$\left\{\begin{array}{c} \theta_{I2}\leq 0\;\;\;\;\;\mathrm{Fault}\;\mathrm{in}\;\mathrm{phase}\;A\\ 0<\theta_{I2}\leq 120\;\;\;\;\mathrm{Fault}\;\mathrm{in}\;\mathrm{phase}\;B\\ 120<\theta_{I2}\leq 240\;\mathrm{Fault}\;\mathrm{in}\;\mathrm{phase}\;C \end{array}\right.$$

Table 4 summarizes the NSC fundamental component angle (*θ*_I2_) for three levels of fault severity under varying load (0%, 50%, and 100%) and frequency (10 Hz, 30 Hz, and 50 Hz) combinations.

For each case shown in Table 4, the recorded values of the *I*_2_ and *θ*_I2_ are derived from three independent observations (N = 3 each operational point). A typical outlier-rejection strategy, the 3-σ rule, was utilized to ensure statistical reliability and mitigate the impact of measurement noise or transient disruptions. Any data point over three standard deviations from the mean throughout the three trials was eliminated and substituted with a repeated measurement to ensure a consistent sample size. No outliers were detected in this dataset, affirming strong repeatability. The strength of the phase-localization criteria in Equation (21), which categorizes faulty phases according to *θ*_I2_ angle ranges, is thus reinforced by minimal intra-condition variation and the lack of outliers across all measured conditions.

The experimental results shown in Table 4 demonstrate a steady increase in the amplitude of the NSC component as the mechanical stress increases, despite a constant fault severity. This tendency is notably apparent across all fault levels and supply frequencies. This behavior in ITSC faults can be explained in two principal processes. First, as the load intensifies, the motor consumes a higher fundamental current, which intrinsically results in increased fault current flow within the shorted turns. Secondly, and more importantly, the increased load results in more magnetic saturation within the stator core, particularly in the defective phase. This localized saturation alters the flux distribution, exacerbates the phase imbalance, and consequently amplifies the negative-sequence component’s amplitude. The nonlinear magnetization response under faulty and loaded conditions enhances the asymmetry induced by the ITSC. The increase in *I*_2_ under load is not solely due to elevated phase current but also stems from saturation-induced asymmetry, which is crucial for fault observability and diagnostic sensitivity under practical operating settings.

## 4. Discussion

This section evaluates the accuracy of the proposed fault detection method under several operating scenarios and compares it with traditional techniques. The focus is directed towards detection accuracy, dynamic response, and robustness in non-ideal disturbances.

The main objective of this study was to create and verify a fault detection algorithm appropriate for inverter-driven IMs. The experimental verification, conducted in a controlled laboratory setup, utilized test conditions—such as load types, inverter topologies, and motor parameters—that were intended to replicate standard industrial conditions. Nevertheless, we recognize that the laboratory setup does not entirely duplicate the complicated dynamics of real industrial scenarios, including variable climatic influences, electrical interference, and mechanical disruptions. This limitation may influence the method’s resilience and generalizability in industrial applications. To resolve this, we recognize the necessity for further testing in active industrial environments. Future work will entail implementing the proposed methodology in an actual system in a factory to evaluate its reliability under more varied and unpredictable circumstances. This testing will quantify the method’s sensitivity and specificity amongst real-world noise and disruptions, hence enhancing the technique’s practical usability.

In contrast to the conventional FFT-based NSC component analysis, the proposed FDI method exhibits enhanced diagnostic accuracy across all studied fault severities, load conditions, and supply frequencies. Table 4 illustrates that the FFT-based *I*_2_ escalates with fault severity; however, there is significant overlap among varying severity levels. For example, at 50% load and 30 Hz, *I*_2_ measures 0.28 A for a minor fault (*μ* = 0.01) and 0.46 A for a moderate fault (*μ* = 0.07), resulting in a mere 0.18 A difference, which may be obscured by system variability. Furthermore, in no-fault conditions, residual asymmetries and magnetic imbalance can produce *I*_2_ values ranging from 0.12 A to 0.18 A, resulting in false-alarm probabilities of up to 45% when a fixed threshold of about 0.12 A is utilized. On the other hand, the proposed FDI demonstrates a significantly higher gradient with fault severity (Table 2). For instance, the FDI increases from 0.16 to 0.51 at severity *μ* = 0.01 and from 0.57 to 0.84 at severity *μ* = 0.15, exhibiting no overlap, which facilitates distinct thresholding at 0.13 (Table 3) and minimal false alarms throughout 27 examined cases. This greatly improves early-stage fault detectability, especially in low-load and low-speed conditions when FFT-based *I*_2_ detection is ineffective due to inadequate signal resolution. Furthermore, the FDI demonstrates superior frequency robustness: for instance, at 10 Hz, a severity of *μ* = 0.01 results in an FDI value of 0.16, whereas *I*_2_ equals 0.12 A, which falls below the majority of practical detection thresholds. The proposed method demonstrates a sensitivity increase of 100%, eliminates false alarms in trials, and enhances diagnostic accuracy. In contrast, the FFT-based *I*_2_ is characterized by an unreliable threshold, inadequate fault severity discrimination, and susceptibility to load-induced magnetic saturation artifacts. Consequently, the FDI offers a more precise, noise-resistant, and load-invariant detection mechanism for ITSC faults in inverter-fed IMs.

Table 5 demonstrates that the proposed technique reaches a detection accuracy of 99% compared to the evaluated negative-sequence-based algorithms. This exceptional performance is due to its strong normalization and weighting mechanism, which increases sensitivity to ITSC-induced asymmetries while mitigating harmonic-related interferences. Conversely, systems like PCA-NSC [38] provide comparable accuracy, but are constrained by computing demands, whereas conventional compensation [15] and impedance-based methods [37] experience diminished accuracy under harmonic distortions or varying operating conditions.

Table 6 demonstrates that the proposed method achieves a notably faster transient response, exhibiting an execution time of roughly 81 μs and a detection delay of under 10 ms on an Intel i5 (2.5 GHz) processor. In contrast to the conventional NSC-based technique, which registers 108 μs and approximately 35 ms, this demonstrates a 25% enhancement in computing efficiency and a 71% decrease in detection latency, facilitating dependable sub-cycle fault detection in real-time applications.

The resilience of the proposed fault detection approach to non-ideal operating conditions—such as slight voltage or current imbalances, electrical noise, and modest fluctuations in load or rotor speed—has been incorporated into the algorithm design. The application of a normalized NSC, along with the optimized weighting coefficient, reduces the impact of such disturbances by diminishing non-fault-induced asymmetries. Consequently, the method preserves elevated detection accuracy while avoiding false alarms in the presence of minor imbalances or transitory fluctuations, which are characteristic of practical inverter-fed IMs. Preliminary experimental trials have validated its stable performance in these scenarios; however, a comprehensive robustness validation involving controlled injection of artificial noise and imbalances is scheduled for future work to further confirm the method’s reliability in practical industrial applications.

The main contribution of the proposed method is its incorporation of a normalized NSC with an experimentally optimized weighting coefficient, which improves sensitivity to ITSC faults while ensuring resilience against inverter-induced harmonics, load transients, and voltage imbalances. The proposed FDI differs from traditional NSC-based approaches, which depend on preset thresholds or model-specific compensation, by dynamically adjusting to baseline asymmetries and functioning efficiently in real-time with minimal computational complexity. The balance of accuracy, rapidity, and straightforwardness distinguishes it from spectral analysis techniques, which frequently experience delays and substantial processing requirements, as well as from model-based observers, which are susceptible to parameter uncertainties. The method provides a lightweight and effective solution for defect detection in inverter-driven IMs under varying industrial settings.

The feasibility of the proposed detection method for high-power IMs and industrial-scale applications is substantiated by its minimal computational complexity and dependence on readily observable electrical parameters. The method relies exclusively on stator current measurements, which are often accessible in industrial motor drives, allowing for effortless integration into existing monitoring systems without necessitating hardware alterations or invasive sensors. Moreover, the fundamental algorithm relies on basic linear operations and normalization, facilitating real-time execution even on inexpensive embedded processors or digital signal controllers (DSCs), commonly employed in industrial settings. The current validation, conducted on a low-power test motor, is based on scalable principles, and the technique is applicable as long as three-phase current signals are accessible. Subsequent work will encompass field-level evaluations of medium- and high-voltage motor systems to further validate scalability and performance under authentic industrial loads and environmental conditions.

## 5. Conclusions

This paper presents an effective and reliable framework for the detection and localization of ITSC faults in IMs functioning under variable speed and load conditions. The methodology focuses on an FDI based on the normalized magnitude of an NSC, which acts as a sensitive measure of stator asymmetries caused by ITSC faults. An interpolation-based threshold-setting technique was utilized to facilitate robust, condition-independent diagnostics. A universal detection threshold was developed by interpolating FDI values across experimentally recorded fault severity levels, hence avoiding the necessity for case-specific adjustments or external condition modeling.

In addition to fault identification, the framework incorporates phase-angle analysis of the essential NSC component to ascertain the faulty phase. The observed angular changes systematically vary with the fault’s position, facilitating fault detection without the need for additional sensors or intricate signal injection. The integration of FDI and NSC phase angle produces an inexpensive, sensorless diagnostic framework appropriate for real-time application in industrial motor monitoring systems.

## Figures and Tables

**Table 1:** Parameters of main equipment for experimental setup.

Parameter	Symbol	Typical Value	Unit
Fault severity factor	*μ*	0.01–0.15	pu
Resistance degradation factor	*α_f_*	1.5–3	-
Inductance reduction factor	*β_f_*	0.8–1.2	-
Fault progression rate	*γ_f_*	0.05–0.2	-
**Induction motor**	*YS-7124*	-	-
Motor rated power	*P*	0.37	kW
Rated rotor speed	*n_r_*	1400	rpm
Stator resistance	*R_s_*	0.5	Ohm
Rotor resistance	*R_r_*	0.3	Ohm
Stator inductance	*L_s_*	2.5	mH
Rotor inductance	*L_r_*	2.0	mH
Mutual inductance (stator–rotor)	*L_m_*	150	mH
**Inverter**	*INOVANCE MD600S*	-	-
Base electrical frequency	*f_e_*	50	Hz
DC bus voltage	*V_dc_*	650	V
Switching frequency	*f_sw_*	5	kHz
**Tension controller**	*HD800*	-	-

**Table 2:** FDI for different fault severities under varying load and frequency conditions.

Fault Severity	Load	FDI		
		280 rpm (10 Hz)	840 rpm (30 Hz)	1400 rpm (50 Hz)
0.01	0%	0.16	0.18	0.32
	50%	0.25	0.25	0.37
	100%	0.39	0.42	0.51
0.07	0%	0.44	0.49	0.54
	50%	0.48	0.52	0.59
	100%	0.54	0.57	0.64
0.15	0%	0.57	0.62	0.65
	50%	0.60	0.67	0.71
	100%	0.74	0.78	0.84

**Table 3:** Summary of FDI for different fault severities.

Fault Severity	Range of FDI	Threshold?	Detection Result
0.01	0.16–0.51	>0.13	High (early-stage fault detection)
0.07	0.44–0.60	>0.13	Very high
0.15	0.57–0.84	>0.13	Extremely high

**Table 4:** Summary of the NSC and its angle under varying fault severity, load, and supply frequency conditions.

Fault Severity	Load	Fundamental Component of NSC (*c*) and Its Angle (*θ_I_*_2_)						Faulty Phase
		280 rpm (10 Hz)		840 rpm (30 Hz)		1400 rpm (50 Hz)		
		*θ_I_*_2_ [°]	*I*_2_ [A]	*θ_I_*_2_ [°]	*I*_2_ [A]	*θ_I_*_2_ [°]	*I*_2_ [A]	
0.01	0%	−81.39	0.12	−81.39	0.18	−81.79	0.24	Phase A
	50%	−81.39	0.21	−82.13	0.28	−82.84	0.34	
	100%	−83.23	0.33	−85.64	0.37	−85.78	0.40	
0.07	0%	158.74	0.38	158.70	0.41	158.32	0.45	Phase C
	50%	158.74	0.42	158.13	0.46	157.48	0.49	
	100%	157.02	0.46	155.26	0.50	155.48	0.53	
0.15	0%	39.27	0.48	38.84	0.51	39.12	0.52	Phase B
	50%	38.66	0.51	37.92	0.54	37.55	0.58	
	100%	36.85	0.64	34.49	0.70	34.83	0.76	

**Table 5:** Comparative analysis of NSC-based detection methods for ITSC faults in IMs.

Ref.	Detection Method	Operating Conditions	Detection Accuracy	Limitations
-	Proposed Method	Inverter-Fed IM under Varying Operational Conditions	99%	-
[15]	Negative-Sequence Current Compensation	Voltage Imbalance Scenarios	93%	May not fully address harmonic distortions
[38]	Negative-Sequence Impedance Analysis	Balanced and Imbalanced Voltages	>90%	Does not address harmonic distortions
[39]	Principal Component Analysis (PCA) with Negative-Sequence Current	Varying Load and Power Quality Issues	>96%	Requires computational resources for PCA
[40]	Harmonic Analysis Using Negative-Sequence Current	Various Levels of Harmonic Distortion and Voltage Imbalance	94%	Comparative study may not cover all fault types

**Table 6:** Real-time performance comparison of the proposed and traditional methods.

Metric	Proposed Method	Traditional NSC-Based Method
Execution Time	~81 μs	~108 μs
Detection Delay	<10 ms	~35 ms

## Data Availability

Data is contained within the article.
